# Book Notices

**Published:** 1883-11-20

**Authors:** 


### *BOOK NOTICES.*

SOUTH-SIDE VIEWS—Dr. Whedon and the Fathers; also, Dr. Haygood's "Our Brother in Black." By Rev. W. J. Scott, North Georgia Conference. Atlanta, Georgia: James P. Harrison & Co., Publishers, 1883. Price, 50 cents.

We have been kindly presented with a copy of the above work by the author, who, for long years we have known and esteemed as a personal friend, and as a gentleman of marked ability as a writer and as a minister of the gospel.

As it is not a medical work and our space is limited, we will not attempt a full review, but recommend our readers to procure the work and read it, especially those who have read Dr. Haygood's book entitled "Our Brother in Black," and his late Chataqua speech touching the education of the negro.

As a critic, Mr. Scott, though fair and liberal, is yet keen, pointed and withering.

In one of the articles in the book he tells us of having provoked the wrath of Dr. Whedon, editor of a Methodist Quarterly, who "without a premonitory word came at him with the snap and snarl of an enraged tom cat." His handling of the said Whedon was tart, cutting and forcible, yet elegant in language.

His criticism upon Dr. Haygood's book and speeches is most timely and well put. His sentiments touching the unity of the races, social equality, negro education, etc., are forcible and true; and he strikes a deep and responsive chord in the Southern heart when he says—"What if, in the unequal strife of the civil war, we were overwhelmed by immense odds, God yet reigns, and the justice or injustice of our cause could not be decided by the arbitrament of the sword. Nor is the future glory of the South to be promoted by truckling sycophancy or unmanly concessions."

